# Diabetic nephropathy; principles of diagnosis and treatment of diabetic kidney disease

**Published:** 2014-01-01

**Authors:** Chaudhary Muhammad Junaid Nazar

**Affiliations:** ^1^Department of Endocrinology, University of Buckingham, Royal Gwent Hospital, NHS Trust, Wales, UK

**Keywords:** Diabetic nephropathy, End Stage renal disease, Diabetes mellitus

## Abstract

Diabetes mellitus is a leading epidemic of the present world. It is considered the leading cause of death among end-stage renal disease (ESRD) patients. The complications associated with diabetes mellitus have boosted the number of deaths in the last years. These complications are the result of long lasting effects of diabetes mellitus on the glomerular microvasculature of the kidney. Diabetic nephropathy (DN) develops in patients with several years’ medical history of diabetes and renal failure. However, research shows that patients with type 1 diabetes progress early to ESRD as compared to those with type 2. DN is more prevalent in ethnic minorities as compared to other groups in society. There are new and different treatment options available since medical science has progressed due to increased research efforts. Unfortunately, there is no permanent cure. The aim of this article is to explore the research of therapeutic strategies currently in use by medical practitioners in order to increase understanding of DN.

**Implication for health policy/practice/research/medical education:**

Diabetes mellitus is a leading epidemic of the present world. It is considered the leading cause of death among end-stage renal disease (ESRD) patients. The complications associated with diabetes mellitus have boosted the number of deaths in the last years. These complications are the result of long lasting effects of diabetes mellitus on the glomerular microvasculature of the kidney. Diabetic nephropathy (DN) develops in patients with several years’ medical history of diabetes and renal failure.

## Introduction

Diabetes mellitus is a leading epidemic of the present world. It is considered the leading cause of death among end-stage renal disease (ESRD) patients. The complications associated with diabetes mellitus have boosted the number of deaths in the last years (1). These complications are the result of long lasting effects of diabetes mellitus on the glomerular microvasculature of the kidney. Diabetic nephropathy (DN) develops in patients with several years’ medical history of diabetes and renal failure. However, research shows that patients with type 1 diabetes progress early to ESRD as compared to those with type 2 DM. DN is more prevalent in ethnic minorities as compared to other groups in society (2). There are new and different treatment options available since medical science has progressed due to increased research efforts. Unfortunately, there is no permanent cure. The aim of this article is to explore the research of therapeutic strategies currently in use by medical practitioners in order to increase understanding of DN.

## Incidence

DN is increasing randomly every year. It is recognised as the most common complication in diabetes patients. Governments spend huge sums for kidney patients every year due to its increasing prevalence. According to the UK prospective study, it is expected to rise by about 25% in the next 10 years (2). In the United States of America, the government devotes $50,000 per year for DN (3). However, the existence ratio of DN is different and variable depending on the cause of occurrence as it is associated with multiple risk factors. Therefore, its existence is different in western countries as compared to the USA (4). Screening programs may help in initial documentation of patients who are at high risk of DN (4).

There are different causes that play an important role in the prevalence of DN. These include genetic variability, lifestyle, diet and different health systems. Therefore, the occurrence of DN differs depending on different causes (5). The highest prevalence of DN is found in native Americans, Mexican-Americans, Asians, Indians and white Europeans (2). The Asian diabetic population is more prone to DN as compared to the western diabetic population due to the presence of microalbuminuria or macroalbuminuria (6).

## Pathophysiology

DN is a clinical syndrome characterised by the insistent albuminuria that should be confirmed on at least two occasions separated by 3-6 months, by continuous decline in the glomerular filtration rate (GFR), and by increased arterial blood pressure. DN is characterised by different events. The characteristic occurrence is thickening of the glomerular basement membrane (GBM). After renal damage, the thickening of the basement membrane starts, which leads to pathologic modifications in mesangial and vascular cells. It includes formation of AGEs, accumulation of polyols, and activation of protein kinase C (7,8). It leads to activation of the inflammatory pathway playing a significant role in the damage of GBM (9).

Secondly, the renal hemodynamic anomaly is similar in both types of diabetes (10). An initial physiologic abnormality is glomerular hyper-filtration related to intra-glomerular hypertension (8,11). This is complemented by the onset of microalbuminuria. Microalbuminuria is considered the first sign indicating the onset of DN (12).

The exact pattern observed in the pathophysiology of DN is:

- Hyperglycaemia
- Thickening of GBM
- Glomerular hyper-filtration
- Impaired endothelial integrity
- Onset of microalbuminuria
- Impairment of nitric oxide transport
- Loss of afferent/efferent auto-regulatory control
- Continued loss of glomerular filtration capabilities

A clinically asymptomatic point of failure follows with development of microalbuminuria (30 mg albumin per day) to macroalbuminuria (>300 mg albumin per day). Once overt nephropathy (macroalbuminuria) has established, renal function falls at a significant but alterable rate (decline in GFR of 2­20 ml/min/year). The rate of decline depends on type of diabetes, genetic predisposition, glycaemic control and, very significantly, blood pressure (12). Hypertension is the single most essential cause of the evolution and opinion of successful intermediation in DN. Later stages may also be supplemented by clinically significant albuminuria, oedema, and nephrotic syndrome. Ultimately, the distinguishing clinical picture of renal failure develops (8). It is thought that the development of nephropathy occurs in similar fashion in both types of diabetes (8).

## Natural clinical course of diabetic kidney disease

The natural history of DN is divided into five stages (2):

Stage 1: Renal pathology develops at the onset of diabetes. The growth of the kidney increases by several centimetres. By the time of diagnosis, the GFR and urinary albumin excretion (UAE) have been increased. It can be controlled at this level by onset of insulin.

Stage 2: The second phase typically lasts for 5-15 years after diagnosis of diabetes. The characteristics of the second phase include:

1. GFR remains elevated due to hyperfiltration.
2. Kidneys remain hypertrophied and UAE rate stays normal.

Stage 3: The characteristics of stage three are:

1. Microalbuminuria is present. It occurs in 30-50% of patients after diabetes onset, 80% of whom go on to develop overt nephropathy over 10-15 years.
2. GFR remains elevated or returns to normal range
3. Blood pressure starts to rise in 60% of patients

Histological changes-progression is as seen in stage two.

Stage 4: This stage is also known as clinical nephropathy or overt nephropathy. The characteristic histological features of stage four are formation of the Kimmelstiel-Wilson nodule (focal glomerular sclerosis) and macroproteinuria. It can progress to nephrotic in 30% of patients or may decline in 80% depending on deterioration of GFR.

Stage 5: As the GFR continues to decline, ESRD may develop. DN is considered the most common cause of ESRD because of associated autoimmune neuropathy and cardiac disease.

The stages of chronic kidney disease (CKD) are mainly based on measured or estimated GFR. There are five stages but kidney function is normal in stage 1 and minimally reduced in stage 2 (Table 1).

**Table 1:** Stages of diabetic nephropathy [modified from the renal association (13)]

**Stage**	**GFR (ml/min/1.73 m** ^2^ **)**	**Description**	**Management**
1	>90	Normal or increased GFR with another evidence of renal damage	Screening CKD and risk reduction
2	60-89	Slightly decreased GFR with another evidence of renal damage	Diagnosis and treatment: slow progression of CKD; comorbidities and cardiovascular disease; risk reduction
3a	45-59	Moderately decreased GFR without evidence of renal damage	Evaluate and treat complication
3b	34-40	Irreversible renal damage	
4	15-29	Severely decreased GFR without evidence of renal damage	Prepare for renal replacement therapy
5	<15	Established renal failure	Renal replacement if uremic

## Screening and diagnosis

In all new patients with diabetes it is imperative to record the previous history of renal diseases or any particular history of hypertension or cardiovascular disease. Urine analysis and correct recording of history of supine or erect blood pressure must be done.

Patients should be screened for microalbuminuria in the diabetic clinic. Albumin is measured as the earliest clinically detectable evidence of DN. Microalbuminuria is a misnomer for albumin in urine. A 24-urine collection is also useful for measuring total protein excretion and creatinine clearance. It is important to note the transient increase in UAE can be caused by uncontrolled hyperglycaemia; or hypertension, fever, urinary tract infection, congestive heart failure or physical exertion (14). Therefore, it is suggested that microalbuminuria should be confirmed by repeating the test of the urine sample over the following 3-6 months. The values have been settled to check the level of risk, which are listed below:

1) Normal: 300 mg, 2) Microalbuminuria: 30-300 mg, 3) Overt proteinuria: >300 mg, 4) Nephrotic syndrome: >3000 mg

The albumin/creatinine ratio (ACR) can be assessed in an early morning demonstration or equally well in random spot urine samples. An ACR of 2.5 is usually taken as the cut-off for microalbuminuria (equivalent to a UAER of >30 mg/24h).

Once a patient develops proteinuria, it is important to rule out causes other than diabetes. This is particularly important in type 1 diabetes. In these cases, patients must be evaluated for other conditions such as hepatitis B and C, human immunodeficiency virus, lupus nephritis, and myeloma, as well as use of non-steroidal anti-inflammatory drugs. Some studies suggest that type 2 patients are at increased risk of developing glomerulopathies. Their diagnoses need to be confirmed by using renal biopsy (14).

The following has been clearly shown in trials using angiotensin-converting-enzyme inhibitors (ACE inhibitors) for type 1 diabetes and angiotensin-II receptor blockers (ARBs) for type 2. These agents also delay time to ESRD in patients with established nephropathy. Comparison with other anti-hypertensive agents suggests that their renoprotective effects are relatively independent of hypertension control.

It is now accepted that blood pressure should be targeted to 125/75 mmHg in patients with stage 4 nephropathy. This will slow, but not stop, the progression of disease in patients with overt nephropathy (14).

The different investigations necessary to perform of patients with DN are listed below (Table 2).

**Table 2:** Investigations for DN

**Urine culture** Exclude infection
**Urine microscopy** Examine for red cell cast in glomerulonephritis
Anti-DNA antibodies
**Complement level** Exclusion of autoimmune disease
Rheumatoid factor
**Igs; Protein electrophoretic strip** Exclude multiple myeloma
**Renal ultrasound** Exclude obstructive renal disease Assess renal anatomy and size

## Management

A lot of research has been done on DN. Treatment of DN should be addressed based on the clinical stage of the disease process. Due to the word count limitation, I will try to focus my discussion on therapeutic prevention of DN and treatment of established nephropathy.

DN can be prevented if diagnosed and managed early by using common prevention methods, including firm control of glucose and blood pressure. Treatment of dyslipidaemia by lifestyle modification changes—including those pertaining to dietary habits, physical activity and weight reduction—is considered a worthy basic intervention for DN patients. Smoking cessation can significantly reduce the cardiovascular risk and must be encouraged. There is evidence that smoking cessation ameliorates progression of microalbuminuria to macroalbuminuria and improves renal prognosis (15). Studies on type 1 diabetes show that firm glycaemic mechanism can reduce the likelihood of patients developing microalbuminuria, and there is emerging evidence that intensive insulin regimes may prevent some microalbuminuria patients from progressing to overt nephropathy (16).

## Specific goals in the prevention of DN

Optimal blood glucose control

1. Control of blood pressure at 120/70 mmHg
2. Avoidance of potential use of nephrotoxic drugs such as nonsteroidal Antiinflammatory Drugs (NSAIDs), aminoglycosides, *etc*.
3. Early detection and management of diabetes, especially in setting of family history.

## Glycemic control

It is a well-established fact that poor metabolic mechanism is a serious risk factor in the aetiology of DN. The studies favouring the early control of glucose level were mostly performed on patients with normal albuminuria or in the early stage of DN. Other studies addressing intensive blood glucose control in advanced kidney disease produced unreliable results as they are confounded by the presence of other comorbid conditions, for example cardiovascular disease (CVD) and hypertension. There is evidence to support glycaemic control in reducing the risk of worsening albuminuria and renal functional decline in diabetes type 1 with overt DN (17,18). This is debatable as research is ongoing into what extent glucose toxicity itself is directly causative in the renal lesion. At the very least, glucose is a significant and clinically relevant marker for the metabolic abnormality that leads to nephropathy, as shown in the diabetes control and complications trial (DCCT) and other treatment trials that exhibit decreased nephropathy with lowered serum glucose (19).

In type 2 diabetic patients associated with DN, strict glycemic control may increase renal histology but does not provide protection against macrovascular complications (20). The most appropriate target for glycated hemoglobin for patients with DN is 7.0%, especially for high-risk patients with established cardiovascular disease. Recently, the ADVANCE study inveterate the expected reductions in new onset microalbuminuria and nephropathy in patients with nearly normal glycaemic control (HbA_1c_ of 6.5% versus 7.3%). However, tense glycaemic control in patients with advanced kidney disease has failed to lower the rate of cardiovascular events (21). The American diabetes association and the European association for the study of diabetes guidelines recommend lifestyle modification first and then propose the addition of basal insulin, sulfonylurea and thiazolidinediones if HbA_1c_ still exceeds in the initial stages of DN (6).

## Blood pressure control

Control of blood pressure undoubtedly offers the best chance of delaying ESRD. The role of ACE inhibitors in the prevention of DN in type 1 diabetic patients has not been defined. In type 1 diabetic patients with microalbuminuria, ACE inhibitors reduce the risk of progression to overt nephropathy (22,23). A collaborative study group trial demonstrated that in patients with macroalbuminuria or overt nephropathy, captopril reduced albuminuria and GFR and delayed the onset of kidney failure compared with a placebo (12,24). Whereas in type 2 diabetes, there is more information available on the renoprotective effect of ARBs compared with ACE inhibitors. In the IRMA 2 study, ARB irbesartan reduced progression to overt nephropathy by 70% in hypertensive type 2 diabetic patients during a 2-year follow-up period (25).

In patients with type 2 diabetes, ACE inhibitors and ARBs both diminish the risk for DN and reduce the occurrence of cardiovascular events. ACE inhibitors and ARBs have been used simultaneously for therapeutic synergy in patients with non-diabetic renal disease (COOPERATE trial) (26).

There are many other drugs used as antihypertensive agents in DN, including diuretics, calcium channel blockers, beta-blockers and direct renin inhibitors (aliskiren) (16). Calcium channel blockers (e.g. diltiazem, verapamil) have been shown in studies to demonstrate antiproteinuria effects (27). But a few agents (e.g. nisoldipine, nifedipine, amlodipine) seemed to play no role in reducing albuminuria or the level of renal disease (28).

## Established nephropathy and cardiovascular risk

Patients diagnosed with DN and with constant proteinuria are at high risk of developing cardiovascular complications. Therefore, courtesy must be paid to control the curable risk factors. Smoking must be discouraged. Diabetes and blood pressure must be controlled to prevent complications up to possible limits. The lipid profiles of DN patients are unbalanced. The combination of reduced high-density lipoprotein (HDL) cholesterol and raised triglycerides and LDL cholesterol contributes to the high prevalence of macrovascular disease (8,12). Statins are considered as the treatment of choice for this scenario because they are metabolised by the liver (Box 1).

**Box 1. Reducing cardiovascular risk factors [Modified table from NICE guidelines (14)]**

- Use statins for the primary prevention of cardiovascular disease in the same way as for people without CKD
- Offer statins for the secondary prevention of cardiovascular disease irrespective of baseline lipid values
- Offer antiplatelet drugs for the secondary prevention of cardiovascular disease. Low dose aspirin can be used but there is increased risk of minor bleeding in people with CKD who are given multiple antiplatelet drugs

## Dyslipidemia

Most patients with DN have dyslipidaemia, characterised by low levels of HDL cholesterol, high triglycerides (TG levels), and a shift from larger towards smaller LDL cholesterol (29). Dyslipidaemias in diabetic patients may contribute to the development of glomerulosclerosis and progressive renal disease. Treatment with statins of patients with type 2 diabetes and patients with non-dialysis-dependent DN provides significant cardiovascular benefit (30,31). Current guidelines recommend a goal for LDL cholesterol of below 100 mg/dl for diabetic patients in general and below 70 mg/dl for diabetic patients with CVD (11).

## Renal biopsy

Renal biopsy is considered in patients with renal disease but without diabetic retinopathy. It is important for those patients with rapidly progressive renal disease—for example when red cell casts seen on microscopy may indicate active glomerulonephritis (32). However, there are some clinical features that help in making the decision to do renal biopsy, summarised as:

1) Short-duration type 1 diabetes
2) Autoimmune disease
3) Mild or absent retinopathy
4) Red cell casts in urine
5) Significant and persistent proteinuria

## Potential new therapies

There is a lot of research ongoing regarding the treatment of DN:

1. High doses of thiamine and its derivative benfotiamine have been shown to reduce the rate of microalbuminuria in experimental diabetes nephropathy, probably due to the decrease in activation of protein kinase C, protein glycation and oxidative stress (3).

2. Treatment with a protein kinase C beta inhibitor normalises GFR, decreases albumin excretion rate, and ameliorates glomerular lesions in diabetic rodents (33).

3. Pimagedine, a second-generation inhibitor of advanced glycation end product, reduced urinary protein excretion and the decline in GFR in proteinuric type 1 diabetic patients in a randomized, placebo-controlled study (34).

## Mortality risk and DN

In both types of diabetes, persistent proteinuria is a common finding. Long-term studies show that the life expectancy of type 1 diabetic patients is approximately 10 years after the onset of proteinuria, with two-thirds of deaths related to end stage renal failure (ESRF) and one-third to cardiovascular disease. In type 2 diabetes, due to the presence of comorbid conditions and cardiovascular disease in those with overt nephropathy, the risk of mortality is so extraordinary that many die before they reach end-stage renal failure (ESRF) (2). According to NICE guidelines, the following patients should be referred early to the specialist (34):

1) Stage 4 and 5 CKD (with or without diabetes)
2) Proteinuria together with hematuria
3) Rapidly declining GFR
4) Hypertension that remains poorly controlled despite use of at least four antihypertensive drugs at therapeutic doses
5) People with, or suspected of having, rare or genetic causes of CKD.

## Conclusion

The mortality of DN patients is extremely high. For example, patients with type 1 diabetes have a mortality 20 times greater than that of the general population, and this relative risk may be magnified by a further 25 times for those with proteinuria (e.g. 2 years mortality for 30% of patients with ESRD). The mortality of DN patients is largely the result of comorbid cardiovascular disease. Most patients with stage 4 DN die before they reach ESRF (35).

In the last few years, we have witnessed great progress in the understanding of the risk factors and mechanisms of DN, the stages of renal involvement in diabetes, and the treatment strategies to prevent or disrupt the progression of DN. Early detection of DN, adoption of multifactorial interventions targeting the main risk factors (hyperglycaemia, hypertension, dyslipidemia and smoking), and use of agents with a renoprotective effect (ACE inhibitors and/or ARBs) do indeed slow the progression of renal disease. Treatment of hypertension is a priority. Attention to these procedures will also ensure the reduction of cardiovascular mortality.

## Author’s contribution

CMJN was the single author of the paper.

## Ethical considerations

Ethical issues (including plagiarism, misconduct, data fabrication, falsification, double publication or submission, redundancy) have been completely observed by the author.

## Conflicts of interests

There were no points of conflicts.

## Findings/supports

None.
